# Interleukin 6 and interferon gamma haplotypes are related to cytokine serum levels in dogs in an endemic *Leishmania infantum* region

**DOI:** 10.1186/s40249-023-01058-3

**Published:** 2023-02-10

**Authors:** Luis Álvarez, Pablo-Jesús Marín-García, Pilar Rentero-Garrido, Celia Pilar Martinez-Jimenez, Lola Llobat

**Affiliations:** 1 Department of Animal Production and Health, Public Health and Food Science and Technology, Veterinary Medicine Faculty, Facultad de Veterinaria, Universidad Cardenal Herrera-CEU, CEU Universities, Valencia, Spain; 2 Precision Medicine Unit, INCLIVA Biomedical Research Institute, Valencia, Spain; 3 Helmholtz Pioneer Campus (HPC), Helmholtz Zentrum München, Neuherberg, Germany

**Keywords:** Cytokine, Haplotype, Dog, Immune response, *Leishmania*, Regulatory mechanism, Resistance, Susceptibility

## Abstract

**Background:**

The Ibizan Hound is a canine breed native to the Mediterranean region, where leishmaniasis is an endemic zoonosis. Several studies indicate a low prevalence of this disease in Ibizan Hound dogs, whereas other canine breeds present a high prevalence. However, the underlying molecular mechanisms still remain unknown. The aim of this work is to analyse the relationship between serum levels of cytokines and the genomic profiles in two canine breeds, Ibizan Hound (resistant canine breed model) and Boxer (susceptible canine breed model).

**Methods:**

In this study, we analyse the haplotypes of genes encoding cytokines related to immune response of *Leishmania infantum* infection in twenty-four Boxers and twenty-eight Ibizan Hounds apparently healthy using CanineHD DNA Analysis BeadChip including 165,480 mapped positions. The haplo.glm extension of haplo.score was used to perform a General Linear Model (GLM) regression to estimate the magnitude of individual haplotype effects within each cytokine.

**Results:**

Mean levels of interferon gamma (IFN-γ), interleukin 2 (IL-2) and IL-18 in Boxer dogs were 0.19 ± 0.05 ng/ml, 46.70 ± 4.54 ng/ml, and 36.37 ± 30.59 pg/ml, whereas Ibizan Hound dogs present 0.49 ± 0.05 ng/ml, 64.55 ± 4.54 ng/ml, and 492.10 ± 31.18 pg/ml, respectively. The GLM regression shows fifteen haplotypes with statistically significant effect on the cytokine serum levels (*P* < 0.05). The more relevant are *IL6*-CGAAG and *IFNG*-GCA haplotypes, which increase and decrease the IL-2, IL-8 and IFN-γ serum levels, respectively.

**Conclusions:**

Haplotypes in the *IFNG* and *IL6* genes have been correlated to serum levels of IFN-γ, IL-2 and IL-18, and a moderate effect has been found on *IL8* haplotype correlated to IL-8 and IL-18 serum levels. The results indicate that the resistance to *L. infantum* infection could be a consequence of certain haplotypes with a high frequency in the Ibizan Hound dog breed, while susceptibility to the disease would be related to other specific haplotypes, with high frequency in Boxer. Future studies are needed to elucidate whether these differences and haplotypes are related to different phenotypes in immune response and expression gene regulation to *L. infantum* infections in dogs and their possible application in new treatments and vaccines.

**Graphical Abstract:**

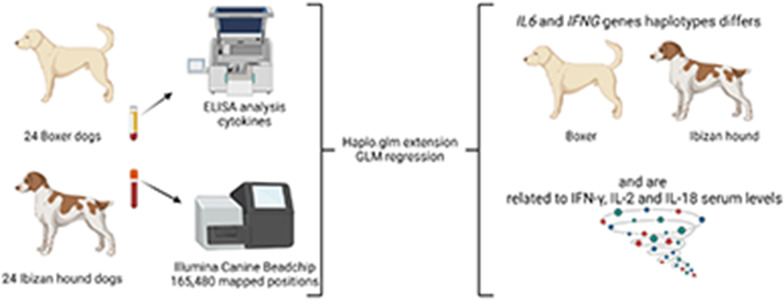

**Supplementary Information:**

The online version contains supplementary material available at 10.1186/s40249-023-01058-3.

## Background

Leishmaniasis is a vector-borne zoonotic disease caused by infection with the obligate intracellular protozoan parasite in the family Trypanosomatidae, order Kinetoplastida, genus *Leishmania* [1], which is transmitted by phlebotomine sandflies from the Psychodidae family [2]. This disease could present with different clinical manifestations, which are classified into mucocutaneous (ML), cutaneous (CL) or visceral (VL) form (kala-azar disease), the latter being the most pathogenic and caused by *L. donovani* in Asia and Africa, and by *L. infantum* in the Mediterranean Basin, the Middle East, Central Asia, South America and Central America [3]. The VL form causes around 20,000 to 40,000 deaths in humans, with 200,000 to 400,000 new infections per year, being one of the most relevant parasitic diseases [4]. Although the parasite has recently been found in different species including reptiles [5], wild carnivores [6, 7], wild rabbits [8, 9], horses [10] and cats [11], the most relevant host of *L. infantum* is the dog, where it causes canine leishmaniasis (CanL) [12].

Seroprevalence of *L. infantum* infection is related to different factors, with controversial results. For example, Gálvez et al. and Rombolà et al. found higher prevalence in males than females, and in younger dogs than older ones, whereas Varjao et al. found no association between seropositivity and sex, but described a higher prevalence in older dogs than younger ones [13–15]. Different seroprevalence related to canine breed is commonly cited in several papers, with higher prevalence in Doberman Pinscher or Boxer when compared to the autochthonous canine breeds of endemic areas [14, 16]. One of them is the Ibizan Hound, an autochthonous canine breed of the Balearic Islands, which appears to be resistant to *L. infantum* infection compared to other breeds [17]. In fact, the study conducted by Solano-Gallego et al. showed a significant cellular response to infection in this canine breed [18]. Cellular response mediated that Th1 response is related to the production of several cytokines, specifically interferon gamma (IFN-γ), tumour necrosis factor alpha (TNF-α) and interleukin 2 (IL-2), which activate the macrophage that eliminates the parasite [12, 19]. Other cytokines such as IL-4, IL-10 and transforming growth factor beta (TGF-β) activate the Th2 response (humoral immune response) and lead to dissemination of the parasite [20]. Abbehusen et al. [21] related *L. infantum* infection with CXCL1 production, which produces a cellular immune response and increases levels of several cytokines such as IFN-γ, IL-6 and IL-18, whereas TNF-α, IL-2 and IL-8 levels are decreased. Thus, canine breeds resistant to infection could present different levels of cytokines other than IFN-γ, which triggers the Th1 response. Furthermore, the genetic factors associated with cytokine levels and resistance to *L. infantum* infection have not been studied. A few studies have been carried out in this sense, but none of them related to the Ibizan Hound. Specifically, twenty-four polymorphisms have been analysed in the *SLC11A1* gene in 40 dogs of different canine breeds, and two of them were associated with increased risk for CanL [22]. The *SLC11A1* gene is related to autoimmune and infectious diseases in humans, such as fibrosis progression in hepatitis C, Crohn’s disease, type-1 diabetes mellitus and tuberculosis [23–26]. In *L. infantum* infection, this gene controls the replication of intracellular parasites [27], and its haplotypes TAG-9-145 and TAG-8-141 are found more frequently in Boxer dogs than other canine breeds and, given that the CanL is elevated in this breed, the authors conclude that this gene could be related to leishmaniasis susceptibility [28]. Quilez et al. [29] conducted a genome-wide association study in the same canine breed and found a region in chromosome 4 which could present several markers with the greatest effect on the susceptible phenotype. However, none of these studies have been able to relate the genetic differences found within the different levels of cytokines in breeds described as resistant or susceptible to the disease.

The aim of this work is to analyse the relationship between serum levels of cytokines and the genomic profiles in two canine breeds, Ibizan Hound (resistant canine breed model) and Boxer (susceptible canine breed model).

## Methods

### Animals and epidemiological data

Information on thirty-one Boxer and twenty-eight Ibizan Hound dogs was recorded from animals living in the Valencia Community (Eastern Spain, Mediterranean region). Data and samples were recovered from October 2021 to June 2022. Apparently healthy dogs which had never presented clinical signs were tested for anti-*Leishmania* specific immunoglobulin G (IgG) antibodies by indirect immunofluorescent antibody test (IFAT) (MegaFLUO^®^ LEISH, Megacor Diagnostik GmbH, Hörbranz, Austria). Only animals with IFAT titre < 1/80 were considered seronegative [30] and included in this study. Seven Boxer dogs were positive for IFAT test and excluded from the study. The epidemiological data of animals included are shown in Table 1.

**Table 1:** Epidemiological data of Boxer and Ibizan Hound dogs analysed

Variable	Categories	Number of dogs (%)	
		Boxer	Ibizan Hound
Gender	Male	13 (54.2)	17 (60.7)
	Female	11 (45.8)	11 (39.3)
Age	Puppy (< 1 year)	3 (12.5)	2 (7.1)
	Young (1 to 5 years)	8 (33.3)	6 (21.4)
	Adult (5 to 10 years)	11 (45.8)	9 (32.1)
	Elder (> 10 years)	2 (8.3)	11 (39.3)
Diet	Only commercial food	24 (100.0)	23 (82.1)
	Home prepared/raw food consumption	0 (0.0)	5 (17.7)
Overall		24 (100.0)	28 (100.0)

The epidemiological variables are gender (male or female), age (puppy-less than one year old, young-between one to five years old, adult-between five to ten years old, and elder,-more than ten years old), and diet (fed only commercial feed or not)

### Sample collection and cytokine levels

Ten millilitres of whole blood were taken by cephalic venipuncture with Vacutainer tubes without anticoagulant. Samples were kept at room temperature to obtain serum aliquots, which were stored at – 20 °C until processing. The whole blood samples were used for DNA isolation within 24 h of extraction.

The IL-2, IL-6, IL-8, IFN-γ [Canine IL-2 ELISA kit, Canine IL-6 ELISA kit, Canine IL-8 ELISA kit, and Canine IFN-γ ELISA kit, Invitrogen (Waltham, Massachusetts, USA), respectively], and IL-18 (Canine IL-18 ELISA kit, Mybiosource, San Diego, CA, USA) levels were measured in serum samples using a commercial ELISA method kit following the manufacturer’s recommendations. In brief, a 100 µl sample of serum was used for analysis with the sandwich-ELISA. The microplate had been pre-coated with an antibody specific to cytokines. The sample was added to the microplate wells and combined with the specific antibody. Then, a biotinylated detection antibody specific for each cytokine and Avidin-Horseradish peroxidase (HRP) conjugate were added successively to each microplate well and incubated. Free components were washed away. The substrate solution was added to each well. The enzyme-substrate reaction was determined by optical density (OD) and measured spectrophotometrically at a wavelength of 450 nm in the plate reader Victor-X3™ (Perkin Elmer^®^, Waltham, Massachusetts, USA). The concentration of each cytokine was calculated by comparing the OD of the samples to the standard curve.

### DNA extraction and whole genome analysis

Genomic DNA (gDNA) from samples was isolated using a QIAamp DNA Blood Kit following the manufacturer’s protocol (QIAamp; Qiagen, Hilden, Germany). DNA was quantified using the Glomax^®^ Discover Fluorimeter and the QuantiFluor^®^ dsDNA kit (Promega, Madison, WI, USA). The genomic DNA concentrations for all samples were a minimum of 50 ng/µl. DNA samples were whole-genome amplified for 20–24 h at 37 ℃, fragmented, precipitated and resuspended in an appropriate hybridisation buffer.

Fifty-two samples (twenty-four Boxer and twenty-eight Ibizan Hound) were genotyped using the CanineHD DNA Analysis BeadChip WG-440-1001 (Illumina, Inc., San Diego, CA, USA) and hybridised on the prepared BeadChips for 16–24 h at 48 ℃. Following the hybridisation, non-specifically hybridised samples were removed by washing, while the remaining specifically hybridised loci were processed for the single-base extension reaction, stained, and imaged on an Illumina iScan Reader (iScan^®^ System, San Diego, CA, USA). GenomeStudio 2.0.5 (Illumina Inc., San Diego, CA, USA) was used to process data generated from the iScan system for subsequent analysis, according to the manufacturer’s guidelines. Intensity data was loaded into the Genotyping Module for primary data analysis, including raw data normalisation, clustering and genotype calling. Single nucleotide polymorphisms on sexual chromosomes and with a call rate < 95% were discarded using PLINK v1.90b6.22 [31]. The final data set includes 165,480 mapped positions in samples with a mean genotyping rate of 0.988.

### Statistical analysis

The homogeneity of epidemiological data of dogs used in the study was evaluated by Fisher’s test analysis. Serum levels of cytokines were analysed using the general linear model procedure (PROC GLM) of the SAS statistical package (version 9.2, North Carolina State University, USA), after normality and homoscedasticity were tested by Shapiro-Wilks and Levene tests, respectively. The model was implemented with sex, age, and breed as fixed effects. Pearson’s correlations between cytokine levels were carried out. The statistical significance was set at* P*-value < 0.05.

Polymorphisms included in each cytokine gene were selected from those genotyped according to the mapping information of the *Canis lupus familiaris* genome assembly CanFam3.1. Upstream and downstream regions (25 kb) were added to each cytokine gene to include possible regulatory regions in the haplotype analysis (Additional file 1: Table S1). PLINK v1.90b6.22 was used to extract variants from the selected genome regions, according to the mapping information of the *Canis lupus familiaris* genome assembly CanFam3.1. The rsID information was downloaded and annotated from the European Variation Archive EVA release 3 files corresponding to the CanFam3.1 assembly.

Haplotypes for each sample were inferred using the haplo.stats version 1.8.9 package in Rstudio [32]. The haplo.stats software computes scores to evaluate the association of a trait with the inferred haplotypes when the linkage phase is unknown. We used the Haplo.glm extension of haplo.score to perform a GLM regression to estimate the magnitude of individual haplotype effects within each cytokine. The haplotypes with an absolute frequency less than 5 were later dropped by setting the haplo.min.count parameter to 5 for the final analysis to only account for major haplotypes.

## Results

**Fig. 1:**
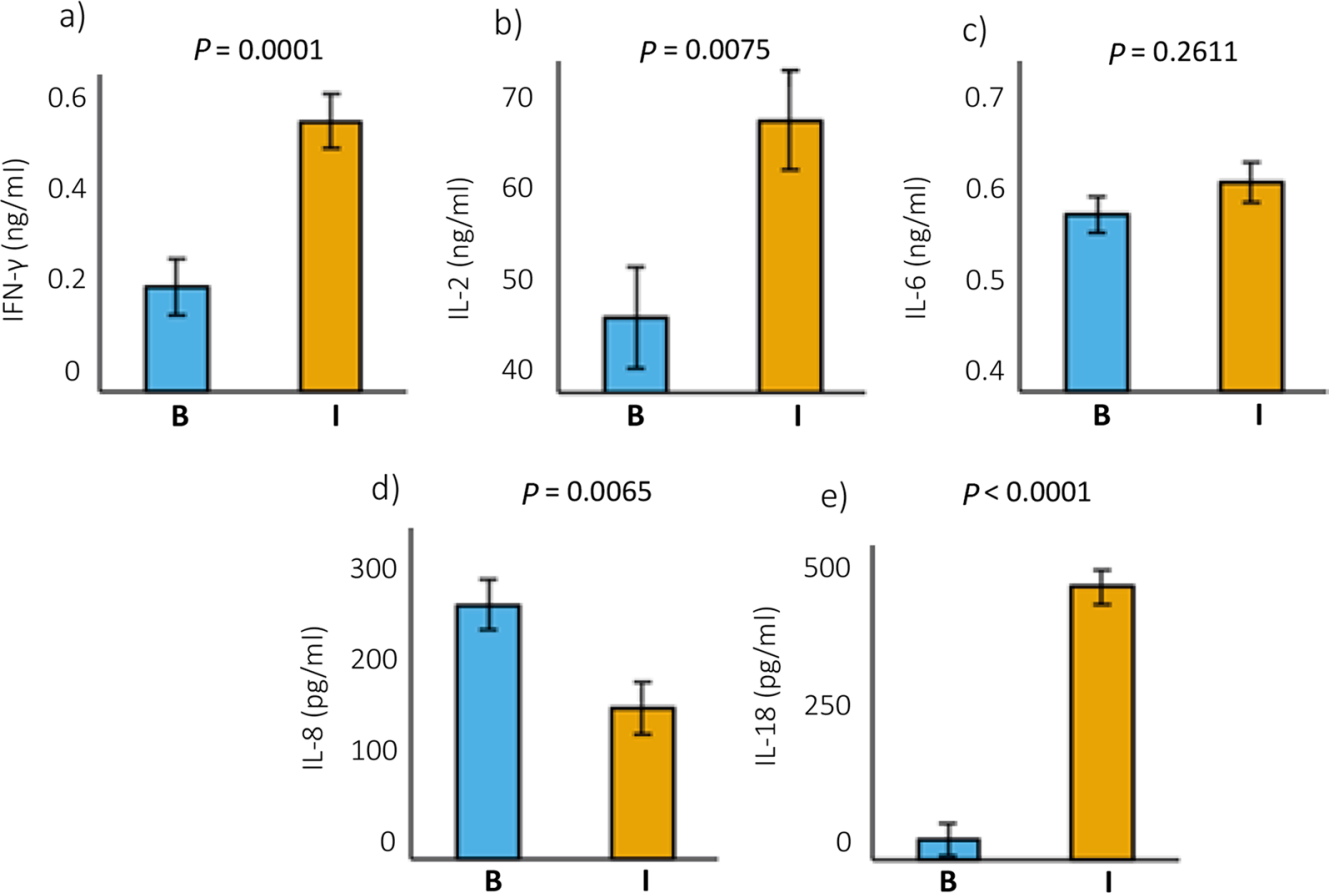
Serum levels of cytokines in Boxer (B) and Ibizan hound (I). Different *P*-values for cytokines are shown in figures **a**–**e**. **a** Interferon gamma (IFN-γ), **b** Interleukin 2 (IL-2), **c** Interleukin 6 (IL-6), **d** Interleukin 8 (IL-8), and **e** Interleukin 18 (IL-18). Squares represent LS means values for two breeds, and vertical lines represent standard deviation. Values for IL-8 and IL-18 are expressed in pg/ml, and for IFN-γ, IL-2, and IL-6 in ng/ml. Different p-values for cytokines are shown in figures **a**–**e**

Differences between cytokine levels were found between the two canine breeds, with IFN-γ, IL-2, and IL-18 levels being higher in Ibizan Hound than in Boxer, whereas IL-8 levels were lower in Ibizan Hound than in Boxer (*P* < 0.05). No statistical differences were found in IL-6 levels between breeds (Fig. 1).

Mean levels of IFN-γ, IL-2 and IL-18 in Boxer dogs were 0.19 ± 0.05 ng/ml, 46.70 ± 4.54 ng/ml, and 36.37 ± 30.59 pg/ml respectively. In contrast, Ibizan Hound dogs present 0.49 ± 0.05 ng/ml, 64.55 ± 4.54 ng/ml, and 492.10 ± 31.18 pg/ml, respectively. Related to IL-8, the values in Boxer dogs are higher than in Ibizan Hounds, at 230.04 ± 23.11 pg/ml and 136.33 ± 23.55 pg/ml, respectively (Table 2).

**Table 2:** Serum levels of cytokines analysed in Boxer and Ibizan Hound

Cytokine^a^	Boxer^b^ (Mean ± SD)	Ibizan Hound^b^ (Mean ± SD)	Mean square	F value	*P*-value
IFN-γ	0.19 ± 0.05	0.49 ± 0.05	1.14	16.95	**0.0001**
IL-2	46.70 ± 4.54	64.55 ± 4.54	4460.00	7.72	**0.0075**
IL-6	0.56 ± 0.09	0.59 ± 0.08	0.01	1.29	0.2611
IL-8	230.04 ± 23.11	136.33 ± 23.55	116,315.75	8.06	**0.0065**
IL-18	36.37 ± 30.59	492.10 ± 31.18	2,750,907.87	108.85	** < 0.0001**

*p*-values < 0.05 are statistically significant and shown in bold

^a^*IFN* interferon; *IL* interleukin

^b^Serum levels of IFN-γ, IL-2 and IL-6 were measured in ng/ml, whereas serum levels of IL-8 and IL-18 were measured in pg/ml

**Fig. 2:**
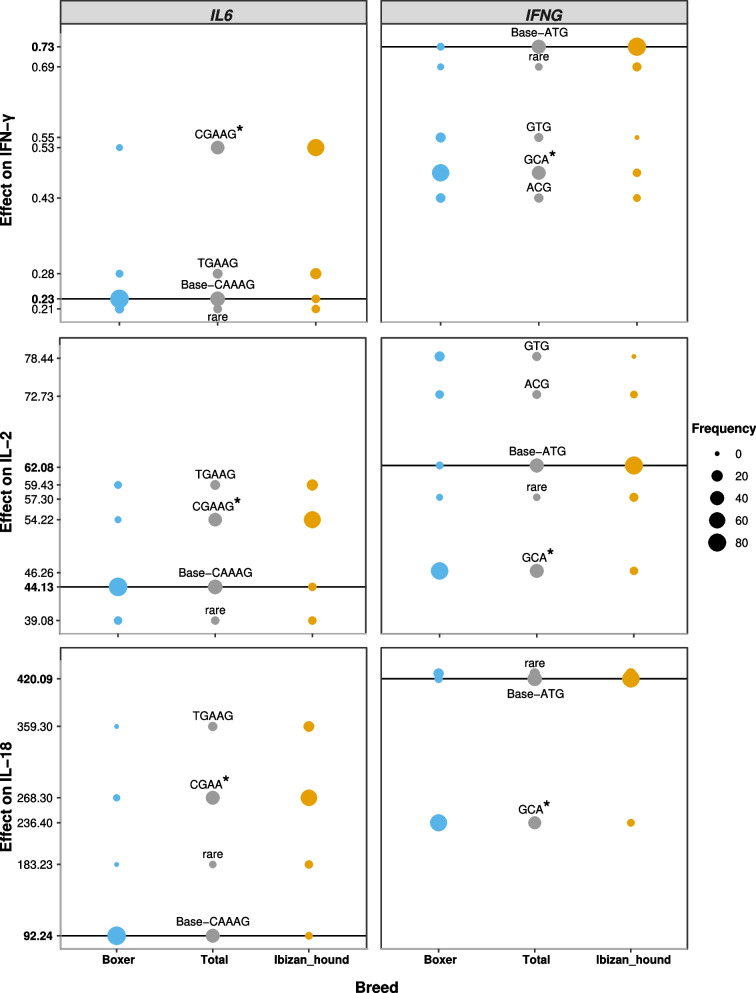
Effects of cytokines haplotype (*IL6*, *IFNG*)-serum levels (IFN-γ, IL-2, IL-18) interactions. Dots represent the expected serum values under the tested GLM for each cytokine haplotype (Predicted values for the basal/base haplotype are signalled with a solid line), sizes are according to their relative frequencies, total and breed specific. Those statistically significant haplotypes are indicated with an *. *IFN* interferon; *IL* interleukin

The complete set of the expected effects of haplotype-serum level interactions under the tested GLM is detailed in Additional file 1: Table S2. We found fifteen haplotypes that presented a statistically significant effect on the cytokine serum levels (*P* < 0.05). These fifteen haplotypes can be divided into two categories: (i) those showing a relative frequency of less than 50% in a specific breed. Thirteen *IL8* haplotypes are involved, which are related to IL-8 and IL-18 serum levels. (ii) Those showing relative frequencies above 50% in a specific breed. There are two haplotypes, *IL6* (CGAAG) and *IFNG* (GCA), which present an extended effect on the IL-2, IL-18 and IFN-γ serum values (Fig. 2).

On the one hand, the *IL6*-CGAAG haplotype, compared against the basal haplotype *IL6*-CAAAG (the most frequent haplotype being > 40%), increases the IL-2, IL-8 and IFN-γ serum levels. Having a single copy of the *IL6*-CGAAG haplotype increases the value of the IL-2 in serum by 10.1 (*P* < 0.05), increases the value of IL-18 by 176.1 (*P* < 0.001) and increases the value of IFN-γ by 0.3 (*P* < 0.01) when compared to a dog being homozygous for the reference haplotype. The *IL6*-CAAAG allele is almost exclusive to the Ibizan Hound breed, with frequencies above 64% in the *IL2*, *IL8* and *IFNG* computed interactions.

On the other hand, the *IFNG*-GCA haplotype that was compared against the reference haplotype *IFNG*-ATG (> 40%) decreases the IL-2, IL-18 and IFN-γ serum levels. Having a single copy of the *IFNG*-GCA haplotype decreases the value of the IL-2 in serum by 15.8 (*P* < 0.001), decreases the value of IL-18 by 183.7 (*P* < 0.001) and decreases the value of IFN-γ by 0.2 (*P* < 0.05) when compared to a dog that is homozygous for the reference haplotype. The *IL6*-CAAAG allele is almost exclusive to the Ibizan Hound breed, with frequencies above 68.5%, while *IFNG*-GCA is almost exclusive to Boxer breed dogs (> 70%) in the *IL2*, *IL8* and *IFNG* computed interactions.

Finally, our results (Additional file 1: Table S2) suggest a moderate effect of *IL8* haplotypes on IL-8 and IL-18 serum levels, which could indicate a relationship between resistance or susceptibility to disease.

## Discussion

The results of the present work show fifteen haplotypes within genes encoding cytokines that correlated with serum cytokines serum levels in apparently healthy dogs of the two canine breeds. Two of them, located in *IFNG* and *IL6,* are statistically correlated with the IFN-γ, IL-2 and IL-18 measured serum levels. The *IL6*-CGAAG are related to increased levels of IFN-γ, IL-2 and IL-18, whereas the *IFNG*-GCA haplotype are related to reduced levels. The frequency of these two haplotypes differs between the two canine breeds, so *IL6*-CGAAG and *IFNG*-GCA haplotypes present high frequency in Ibizan Hound and in Boxer, respectively. Consistent with these results, the Ibizan Hounds present higher serum levels of IFN-γ, IL-2 and IL-18 compared to Boxer dogs.

IFN-γ plays a relevant role in macrophage activation against *Leishmania* infection via nitric oxide [33–35]. When IFN-γ binds its receptor on the cell membrane of macrophages, the JAK-STA-1 pathway is activated, inducing IFN-γ stimulated genes [34]. Furthermore, several studies indicate that IFN-γ regulates the transcriptional mechanisms by alternative splicing and altering the expression of microRNAs and lncRNAs [36]. Regarding *L. infantum,* the control of infection requires the activation of T helper 1 (Th-1) cells, which increases the IFN-γ and IL-2 serum levels [37, 38]. The production of these cytokines was correlated with resistance to disease, so IFN-γ has been proposed as biomarker for immune monitoring in canine leishmaniasis [17, 39, 40], and IL-2 expression was negatively correlated with splenic parasite loads in infected dogs [41]. The IL-18, known as IFN-γ inducing factor, increases the production of this interferon by T cells and has a relevant role in the defence against visceral leishmaniasis [42, 43]. According to that, these three cytokines (IFN-γ, IL-2 and IL-18) present high levels in Ibizan Hound dogs, which have a natural resistance against canine leishmaniasis [18, 39, 44, 45]. Our results indicate that these elevated levels are correlated with the *IL6*-CGAAG haplotype, which is found with a higher frequency in Ibizan Hound dogs compared to Boxers. Different haplotypes of *IL6* gene or *IL6R* genes have been related to IL-6 and other cytokine serum levels in humans, including IL-2, IL-8 and IL-18 cytokines [46–49], and with the severity or protective effect of the infectious disease, including parasitic diseases. For example, Mendonça et al., Sortica et al. and Wujcicka et al. founded *IL6* gene haplotypes related to the severity of the malaria disease and *Toxoplasma gondii* infection [50–52], whereas Chen et al. showed several *IL6* haplotypes with protective effect against COVID-19 infection [53]. According to the results of Yang et al. in human, our results show a correlation between *IL6* haplotype and serum levels of IL-18, in addition to finding an association with high levels of other cytokines such as IFN-γ and IL-2, all of them related to protective effect against *L. infantum* infection.

On the contrary, Boxer dogs present higher frequency of the *IFNG*-GCA haplotype, which is related to low levels of IFN-γ, IL-2 and IL-18. Correlations between *IFNG* gene haplotypes and low level of cytokines have previously been detected in humans. In fact, da Silva et al. demonstrated a correlation between single haplotype of *IFNG* and low levels of IFN-γ, associated with the susceptibility to leishmaniasis [54]. According to these authors, the correlation between *IFNG*-GCA haplotype and the low levels of IFN-γ was also detected, together with low levels of other cytokines with a protective effect in *Leishmania* infection, such as IL-2 and IL-18. The IL-2 cytokine is secreted by the Th1 cells and stimulates the production of IFN-γ [55]. In fact, treatment of exogenous IL-2 in mice reduced parasitic load, increasing the *IFNG* expression [56]. Moreover, the IL-18 cytokine presents a protective effect against visceral leishmaniasis caused by *L. infantum* infection in humans [57], inducing IFN-γ and leading to the production of Th1 responses and natural killer (NK) cells [58].

Although studies correlating haplotypes of *IFNG* gene and cytokine levels and/or susceptibility to infectious disease have not yet been carried out in dog, several studies described different haplotypes in this gene as being related to the susceptibility or resistance to infectious diseases in humans, including parasitic infection diseases. For example, some authors associated *IFNG* haplotypes with the susceptibility to virus infection, such as hepatitis B virus [59], T-lymphotropic virus type 1 infection [60], or tuberculosis [61, 62], as well as bacterial infections such as brucellosis [63], or parasitic infections such as malaria [64, 65]. Related to leishmaniasis, Kalani et al. [66] found several haplotypes in the *IFNG* gene related to susceptibility and resistance to visceral leishmaniasis in Iran.

Several haplotypes of *IL8* gene have been related to susceptibility and severity of different infectious diseases such as tuberculosis [67], syncytial virus disease [68] and hepatitis B [69]. In fact, several studies have demonstrated that *IL8* haplotypes are related to the susceptibility of infectious diseases, increasing the percentage of ROS-producing monocyte-derived macrophages [70] and increasing the influx of neutrophils in inflammatory lesions [71]. However, the relationship between *IL8* haplotype and cytokine serum levels or susceptibility to *Leishmania* infection is still unknown. The limited number of dogs included in our study with these haplotypes made it difficult to obtain determinant results. More studies related to these haplotypes and their effect on the severity of diseases are needed to elucidate the molecular mechanisms of susceptibility and resistance to *L. infantum* in dogs and other mammals, including humans.

## Conclusions

In this study, haplotypes in the *IFNG* and *IL6* genes have been correlated to serum levels of IFN-γ, IL-2 and IL-18, and a moderate effect on *IL8* haplotype correlated to IL-8 and IL-18 serum levels was found. The results indicate that the resistance to *L. infantum* infection could be a consequence of certain haplotypes with a high frequency in the Ibizan Hound dog breed, while susceptibility to the disease would be related to other specific haplotypes, with high frequency in Boxer. Future studies will be necessary to elucidate the specific biological function of these haplotypes, their relationship with cytokine expression and regulation, and with different stages of disease in dogs and other mammals, including humans.

## Supplementary Information

**Additional file 1:** **Table S1.** Analysed poslymorphism in the cytokines genes, including the identification number (rsID), chromosmes (Chr) and genomic position (Pos), according to CanFam3.1 assembly. **Table S2.** Cytokines haplotype-serum interactions. Total haplotype frequencies s are shown along with breed specific values. Coefficients summarized the estimated magnitude of individual haplotype effects. Coefficients values for base haplotypes correspond to the Intercept or constant value in the model.

## Data Availability

All data generated or analysed during this study are included in this published article and its Additional files.

## Abbreviations

CanL: Canine leishmaniasis
GLM: General linear model
IFAT: Indirect immunofluorescent antibody test
IFN: Interferon
IFNG: Interferon gamma
IL: Interleukin
TGF: Transforming growth factor
Th: T helper
TNF: Tumour necrosis factor
VL: Visceral leishmaniasis
